# Inter-dimensional effects in nano-structures

**DOI:** 10.1186/1556-276X-7-581

**Published:** 2012-10-23

**Authors:** Rainer Dick

**Affiliations:** 1 Department of Physics & Engineering Physics, University of Saskatchewan, 116 Science Place, Saskatchewan, S7N 5E2, Saskatoon, Canada

**Keywords:** Density of states, Coulomb interactions, Exchange interactions, Scattering in nano-structures, Thermal properties in nano-structures, Fermi energy in nano-structures

## Abstract

We report on two extensions of the traditional analysis of low-dimensional structures in terms of low-dimensional quantum mechanics. On one hand, we discuss the impact of thermodynamics in one or two dimensions on the behavior of fermions in low-dimensional systems. On the other hand, we use both quantum wells and interfaces with different effective electron or hole mass to study the question when charge carriers in interfaces or layers exhibit two-dimensional or three-dimensional behavior. We find in particular that systems with different effective masses in the bulk and in the interface exhibit separation of two-dimensional and three-dimensional behavior on different length scales, whereas quantum wells exhibit linear combination of two-dimensional and three-dimensional behavior on short length scales while the behavior on large length scales cannot be associated with either two-dimensional or three-dimensional behavior.

## Background

Nano-structures traditionally provide approximate realizations of low-dimensional systems through confined electron states in one dimension (thin films, interfaces or quantum wells), two dimensions (quantum wires or nano-wires), or three dimensions (quantum dots or color centers). We emphasize electron states rather than electrons in the following discussions, because for conduction bands with large filling factors or p-doped semiconductors, we usually think of the unoccupied electron states as holes, which can also be confined [1-3].

In the olden days, confined electron states primarily provided dimensionally restricted realizations of electric charge carriers, but since the advent and rise of spintronics, low-dimensional spin systems also play an important role in nano-technology. Low-dimensional spin systems are directly linked to confined electron states because the coupling of a particle with spin $\vec{S}$ to magnetic fields $\vec{B}$ is proportional to inverse particle mass,

$$H_{I}=-\frac{q}{2m}\vec{B}\cdot \left(\vec{M}+2\vec{S}\right).$$

Here, *m* is the (effective) mass, and *q* is the charge of the particle, *q *= ±*e* for holes or electrons, respectively. $\vec{M}$ is the angular momentum of the particle. Low-dimensional systems in spintronics are therefore directly linked to confined electron states because electrons or holes do not only provide the lightest movable charge carriers, but also interact stronger with magnetic fields than any other readily available (quasi-)particle in materials. Furthermore, energy gaps between different spin configurations are determined by exchange integrals, and the spatial extent of the wave function of a bound particle of mass *m* typically scales with 1/*m*. Exchange interactions between electrons or holes can therefore align or anti-align spins, whereas inter-nuclear exchange interactions are negligible in materials science.

A well-known primary effect of a reduced number *d* of dimensions in nano-systems is the significant change in the energy dependence of the density of states in the energy scale, $\varrho (E)∼{\sqrt{E}}^{d-2}$. This directly affects the thermal and electrical conductivity properties of nano-systems and impacts the use of spins for information storage and processing. Furthermore, even without confinement, particles can exhibit low-dimensional behavior on certain energy and length scales if their propagation properties are affected by the presence of layers, interfaces, or wire-type structures in which the particles propagate with an effective mass which is very different from their effective mass in the adjacent bulk materials [4,5].

**Figure 1:**
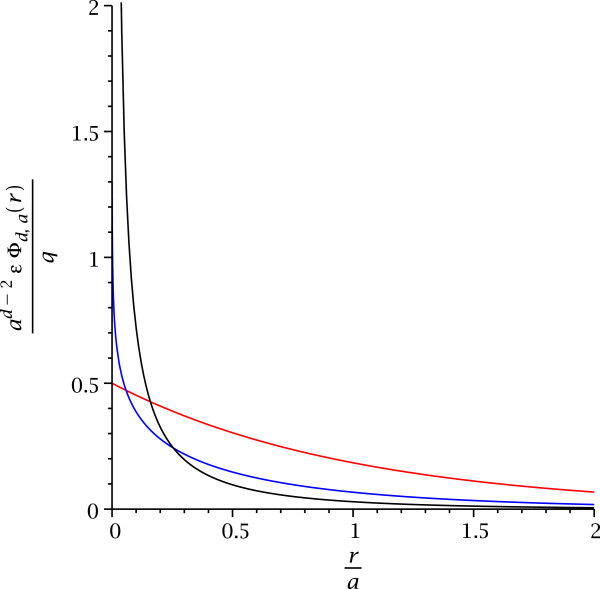
**Electric potentials *Φ***_***d*,*a***_**(*r*) with screening length *a*.** The red curve corresponds to *d*=1, blue to *d*=2, and black to *d*=3.

Confinement of electromagnetic fields and photons is harder to accomplish than for electrons or holes, but effects of restricted dimensionality are also striking and of high potential relevance for technology [6]. Confinement of electromagnetic fields changes the distance law for electric forces to $|\vec{E}(\vec{r})|∼r^{-(d-1)}$ with the potential for logarithmic or linear confinement of electric charges in *d *= 2 or *d *= 1, respectively. Even screened electric forces and potentials are affected, see Figure 1[7].

Figure 1 illustrates that in lower dimensions, interactions are comparatively stronger at large distances and weaker at short distances. The same effect would apply for any other interaction which would be mediated by confined bosons; for example, it would also apply to phonon-mediated interactions between electrons or holes. The reduced interaction strength at smaller distance in lower dimensions is a consequence of the weaker singularity of the field near its source, whereas the increase in strength at larger distance intuitively can be attributed to the squeezing of field lines into a smaller number of dimensions. This change in distance behavior directly impacts electric forces between charges and implies the potential emergence of electrical confinement in systems with dimensionally restricted electromagnetic fields. In addition, it also impacts effective spin-spin interactions in spintronics because the *d*-dimensional electrical potential also appears in the exchange integrals which determine the energy splits between spin configurations.

## Methods

Low-dimensional quantum mechancis with one or two-dimensional Hamilton operators, or three-dimensional Hamiltonians with confining boundary conditions are widely used to analyze and understand the importance of quantum effects on confined particles in nano-systems. Here, we wish to report on extensions of this analysis in two directions: (1) impacts of low-dimensional thermodynamics on the behavior of charge carriers and (2) quantum mechanical analysis of inter-dimensional behavior in materials with a low-dimensional component. We focus also on a thin interface or layer as the low-dimensional component, but the same methods can be applied, e.g., to analyze dimensional competition in the case of a nano-wire on a surface [8]. Inter-dimensional effects in these systems can be relevant, e.g., for charge transport in nano-wires, which attract a lot of interest, e.g., for its use in photovoltaics [9]. We will use both the method of inter-dimensional Green’s functions [4,5,10] and grand canonical ensembles in low-dimensional systems to analyze impact of dimensionality of a system on the behavior of electrons and photons.

The *d*-dimensional fields and potentials are direct consequences of the solutions of Laplace-Poisson or Helmholtz equations in *d* dimensions. The pertinent properties of these solutions are generically encoded in the Green’s functions which satisfy

(1) $$\left(\Delta +\frac{2m}{\hbar^{2}}E\right)\langle \vec{x}|G_{d}(E)|{\vec{x}}^{\prime }\rangle =-\delta (\vec{x}-{\vec{x}}^{\prime })$$

in the energy representation, or

(2) $$\left(i\hbar \frac{\partial }{\partial t}+\frac{\hbar^{2}}{2m}\Delta \right)\langle \vec{x}|G(t)|{\vec{x}}^{\prime }\rangle =\delta (t)\delta (\vec{x}-{\vec{x}}^{\prime })$$

in the time domain. The Green’s functions are related according to

(3) $$\begin{array}{l} \langle \vec{x}|G_{d}(E)|{\vec{x}}^{\prime }\rangle =-\frac{2\text{mE}}{\hbar^{2}}\langle \vec{x}|G_{d}(E)|{\vec{x}}^{\prime }\rangle \\ \;\;\;\;=\int_{-\infty }^{\infty }\;\text{dt}\;\langle \vec{x}|G_{d}(t)|{\vec{x}}^{\prime }\rangle \text{exp}(i\text{Et}/\hbar ), \end{array}$$

(4) $$\langle \vec{x}|G_{d}(t)|{\vec{x}}^{\prime }\rangle =\frac{1}{2\Pi \hbar }\int_{-\infty }^{\infty }\;\text{dE}\;\langle \vec{x}|G_{d}(E)|{\vec{x}}^{\prime }\rangle \text{exp}(-i\text{Et}/\hbar ).$$

The solutions of Equations (1) and (2) provide us with single particle or mean field Green’s functions, which describe scattering of particles and densities of states, and through particular choices or re-definition of the energy parameter, they also determine electric potentials and exchange interactions. In addition, the single particle Green’s functions also enter into the calculation of electronic configurations for many particle systems through application of multiple scattering theory [11].

The conditions (1) and (2) do not completely specify the Green’s functions, and we impose the physical boundary conditions that the Green’s function in the region of negative energy *E *< 0 should vanish for $|\vec{x}-{\vec{x}}^{\prime }|\to \infty$, while the positive energy Green’s function should describe outgoing spherical waves $∼\text{exp}(i\text{kr})/{\sqrt{r}}^{d-1}$, $k=\sqrt{2\text{mE}}/\hbar$, in the limit $r=|\vec{x}-{\vec{x}}^{\prime }|\to \infty$. These conditions yield the well-known retarded Green’s functions

(5) $$\;\begin{array}{l} \langle \vec{x}|G_{d}(t)|{\vec{x}}^{\prime }\rangle =\frac{\Theta (t)}{i\hbar {\left(2\Pi \right)}^{d}}\int \;d^{d}k\text{exp}\;\left[i\;\left(\vec{k}\cdot (\vec{x}-{\vec{x}}^{\prime })\right)\right)\\ \;\;\;\;\;\;\left(\left(-\frac{\hbar }{2m}(t-i\varepsilon ){\vec{k}}^{2}\right)\right]\\ \;\;\;=\frac{\Theta (t)}{i\hbar }{\sqrt{\frac{m}{2\Pi i\hbar (t-i\varepsilon )}}}^{d}\text{exp}\;\left(i\frac{m{\left(\vec{x}-{\vec{x}}^{\prime }\right)}^{2}}{2\hbar (t-i\varepsilon )}\right) \end{array}$$

in the time domain. The energy-dependent Green’s functions are with the notation $\langle \vec{x}|G_{d}(E)|{\vec{x}}^{\prime }\rangle \equiv G_{d}(\vec{x}-{\vec{x}}^{\prime },E)$ given by

(6) $$\begin{array}{ll} G_{d}(\vec{x},E) & =\frac{\Theta (-E)}{{\sqrt{2\Pi }}^{d}}{\left(\frac{\sqrt{-2\text{mE}}}{\mathrm{\hbar r}}\right)}^{\frac{d-2}{2}}K_{\frac{d-2}{2}}\left(\sqrt{-2\text{mE}}\frac{r}{\hbar }\right)\\ & \;+i\frac{\Pi }{2}\frac{\Theta (E)}{{\sqrt{2\Pi }}^{d}}{\left(\frac{\sqrt{2\text{mE}}}{\mathrm{\hbar r}}\right)}^{\frac{d-2}{2}}H_{\frac{d-2}{2}}^{\left(1\right)}\left(\sqrt{2\text{mE}}\frac{r}{\hbar }\right), \end{array}$$

(see Appendix I in [7] for derivations). The functions *K*_*ν *_and $H_{\nu }^{\left(1\right)}$ are modified Bessel functions and Hankel functions of the first kind, respectively, and we follow the notations and conventions of [12] for special functions.

However, if there are parameter ranges in materials and devices where electrons or photons behave according to the laws of two-dimensional or three-dimensional quantum mechanics and electrodynamics, then there should also exist transition regimes with intermittent dimensional behavior. This is the realm where particles or forces are described by the *inter-dimensional* or *dimensionally hybrid* Green’s functions introduced in [4,5]. We should also point out that another important novel approach to inter-dimensional behavior in systems with low-dimensional components concerns the study of *inter-dimensional universality* for critical scaling laws. This notion has been introduced and studied for domain wall dynamics in nano-wires [13].

We will review the basic aspects of physics in various dimensions in the section on “Green’s functions, potentials, and densities of states in *d* dimensions” and then discuss a lesser known but technologically relevant aspect of physics in lower dimensions, *viz.*, the impact of dimensionality on statistical and thermal physics in low-dimensional systems, in the section on “Thermal properties of the charge carriers in *d* dimensions”. We will then discuss the construction of dimensionally hybrid Green’s functions for quantum wells in the section on “Inter-dimensional effects in interfaces and thin layers”. This will also allow us to calculate the inter-dimensional density of states *ϱ*(*E*) and the relation between Fermi energy and electron density in the quantum well in the section entitled “Density of states for the thin quantum well”. Comparison of the results for the quantum well with the results for layers with different effective mass of charge carriers [5] or different permittivity [6] reveals that a difference in *potential energy* between a layer and a bulk yields linear combinations of two-dimensional and three-dimensional terms at the *same* length scales, whereas difference in *kinetic terms* (*viz.* effective mass which affects kinetic terms for electrons, holes, or permittivity, which affects the kinetic terms for photons), *separates* two-dimensional behavior on short length scales from three-dimensional behavior at large length scales.

## Results and discussion

We can now enter into the discussion of less known results on the low-dimensional quantum and statistical physics of charge carriers and new results and observations concerning inter-dimensional behavior in the presence of layers or interfaces. We will separate this discussion into subsections on interaction potentials and thermal properties in low-dimensional fermion systems, and a subsection on inter-dimensional effects as inferred from Green’s functions.

### Green’s functions, potentials and densities of states in *d* dimensions

We have chosen the paradigm of the Green’s functions for the free Schrödinger equation (1,2) because it encompasses most of the practical applications of Green’s functions in materials and devices. The energy-dependent Green’s function for the free Schrödinger equation not only describes the electron or hole scattering of impurities or the density of states in the energy scale in free electron gas models, but it also describes the electric potential of a charge density $\rho (\vec{x})$ and exchange integrals between electron states in *d* dimensions through the zero energy Green’s function

$$\langle \vec{x}|G_{d}(E=0)|{\vec{x}}^{\prime }\rangle \equiv \langle \vec{x}|G_{d}|{\vec{x}}^{\prime }\rangle .$$

The Coulomb and exchange type potentials and interactions are given in terms of this Green’s function through

(7) $$\Phi_{d}(\vec{x})=\frac{1}{\varepsilon }\int \;d^{d}x^{\prime }\;\langle \vec{x}|G_{d}|{\vec{x}}^{\prime }\rangle \rho ({\vec{x}}^{\prime })$$

and

(8) $$\begin{array}{l} J_{nn^{\prime }}=\frac{e^{2}}{\varepsilon }\int \;d^{d}x\int \;d^{d}x^{\prime }\;\Phi_{n^{\prime }}^{+}(\vec{x},t)\Phi_{n}^{+}({\vec{x}}^{\prime },t)\\ \;\;\times \langle \vec{x}|G_{d}|{\vec{x}}^{\prime }\rangle \Phi_{n^{\prime }}({\vec{x}}^{\prime },t)\Phi_{n}(\vec{x},t), \end{array}$$

respectively. Furthermore, with the substitution 2*mE *= −*ℏ*^2^/*a*^2^, the energy-dependent Green’s function also describes screened interaction potentials with screening length *a*,

(9) $$\Phi_{d,a}(\vec{x})=\frac{1}{\varepsilon }\int \;d^{d}x^{\prime }\;\langle \vec{x}|G_{d}(-\hbar^{2}/2ma^{2})|{\vec{x}}^{\prime }\rangle \rho ({\vec{x}}^{\prime }),$$

$$\left(\Delta -\frac{1}{a^{2}}\right)\Phi_{d,a}(\vec{x})=-\frac{1}{\varepsilon }\rho (\vec{x}),$$

and correspondingly screened exchange interactions. Practical realization of low-dimensional Coulomb or Yukawa potentials (Equations (7) and (9) with *d *= 1 or *d *= 2) in devices may be possible with the help of photonic bandgap materials, and the two-dimensional logarithmic behavior should be realized at short distances in high permittivity thin films [6].

However, a more direct and immediate application of *d*-dimensional Green’s functions in materials science and device engineering concerns scattering in low-dimensional structures. Scattering of a particle of momentum $\hbar \vec{k}$ by a localized or screened impurity potential $V(\vec{x})$ is described by a wave function which contains the energy-dependent Green’s function

(10) $$\begin{array}{l} \psi_{\vec{k}}(\vec{x})=\text{exp}(i\vec{k}\cdot \vec{x})-\frac{2m}{\hbar^{2}}\\ \;\;\int \;d^{d}x^{\prime }\;G_{d}(\vec{x}-{\vec{x}}^{\prime },\hbar^{2}k^{2}/2m)V({\vec{x}}^{\prime })\text{exp}(i\vec{k}\cdot {\vec{x}}^{\prime })\\ \;=\text{exp}(i\vec{k}\cdot \vec{x})-\frac{i\Pi m}{{\left(2\Pi \right)}^{d/2}\hbar^{2}}\int \;d^{d}x^{\prime }{\left(\frac{k}{\left|\vec{x}-{\vec{x}}^{\prime }\right|}\right)}^{\frac{d-2}{2}}\\ \;\;\times H_{\frac{d-2}{2}}^{\left(1\right)}\left(k|\vec{x}-{\vec{x}}^{\prime }|\right)V({\vec{x}}^{\prime })\text{exp}(i\vec{k}\cdot {\vec{x}}^{\prime }). \end{array}$$

This yields the differential scattering cross section in *d* dimensions,

(11) $$\frac{d\sigma }{d\Omega }=\underset{r\to \infty }{\text{lim}}r^{d-1}\frac{j_{\text{out}}({\vec{k}}^{\prime })}{j_{\text{in}}(\vec{k})}={\left|f(k\hat{\vec{x}}-\vec{k})\right|}^{2},$$

with the scattering amplitude

(12) $$f(\Delta \vec{k})=-\frac{mk^{(d-3)/2}}{{\left(2\Pi \right)}^{(d-1)/2}\hbar^{2}}\int \;d^{d}x\;\text{exp}(-i\Delta \vec{k}\cdot \vec{x})V(\vec{x}).$$

The most interesting feature of this result from a nano-device point of view concerns suppressed high energy scattering and enhanced low energy scattering from impurities in low dimensions roughly according to $d\sigma /d\Omega ∼k^{d-3}∼{\sqrt{E}}^{d-3}$.

Another application of low-dimensional physics for nano-scale devices concerns the density of states (or number of electronic orbitals) in the energy scale,

(13) $$\varrho_{d}(E)=2\Theta (E){\sqrt{\frac{m}{2\Pi }}}^{d}\frac{{\sqrt{E}}^{d-2}}{\Gamma (d/2)\hbar^{d}}.$$

These are densities of states per *d*-dimensional volume and per unit of energy, i.e., *Vϱ*_*d*_(*E*)*dE *is the number of electronic states in a *d*-dimensional volume *V * and with energies between *E* and *E* + *dE*. The corresponding relation between the Fermi energy *E*_*F *_and the density *n* of electrons in *d* dimensions is therefore

(14) $$n_{d}=\frac{2}{\hbar^{d}\Gamma ((d+2)/2)}{\sqrt{\frac{mE_{F}}{2\Pi }}}^{d}.$$

This makes physical sense: In a smaller number of dimensions, we need a larger Fermi sphere in $\vec{k}$ space to accommodate the same electron density in $\vec{x}$ space.

Equations (13) and (14) *a priori* refer to a free electron gas model. In materials science, this is a useful approximation for semi-conductors and a very good approximation for metals at room temperature. For energy bands with minimal energy *E*_0_, corresponding effective mass *m*_∗_, and a low filling factor, Equation (13) applies for the electron density of states with the substitutions *E *→* E *−* E*_0_ and *m *→* m*_∗_. For nearly filled bands with maximal energy *E*_1_, the substitutions *E *→* E*_1_−* E*, *m *→* m*_*h*_ yield the hole density of states.

Densities of states are important for electrical and thermal transport properties of materials and for the optical properties of materials. For example, the photon absorption cross-section for excitation of an electron from a discrete donor or quantum dot state into a continuous energy band is directly proportional to the density of final electron states. Therefore, the densities of states (13) for *d *= 1, 2, and 3 are common items for information in nano-technology textbooks. However, strict electron confinement to a quantum wire or an interface is apparently a bad approximation in most cases and makes only sense for the subset of low lying energy states in deep quantum well structures. Therefore, we will revisit the density of states in the section on “Density of states for the thin quantum well” in the framework of a solvable quantum well model.

### Thermal properties of the charge carriers in *d* dimensions

A less widely known and less developed aspect of low-dimensional physics concerns the impact of dimensionality of a system on its thermal and statistical properties. The derivation of the basic Fermi-Dirac or Bose-Einstein distributions from maximal information entropy under the boundary conditions of given energy and particle number (if we use a grand canonical ensemble) does not depend on the number of dimensions. However, the calculation of partition functions and thermodynamic quantities from the Fermi-Dirac or Bose-Einstein distributions involves *d*-dimensional integrals; therefore, thermal properties of a system will depend on the number of dimensions in which particles can move. I would hope that the introduction in this section can serve as a brief compendium and overview of basic aspects of this dependence of thermal properties on *d*. We will find that the specific heat in particular is affected by *d*. Due to the particular relevance of confined fermionic charge and spin carriers for nano-technology, we will focus on low-dimensional implications of Fermi-Dirac statistics.

We can derive all the basic properties of the *d*-dimensional fermion gas from its grand canonical potential

$$\;\Omega =-\beta p=-2V\int \frac{d^{d}k}{{\left(2\Pi \right)}^{d}}\text{ln}\;\left(1+\text{exp}\;\left[\beta \mu -\beta E\;\left(\vec{k}\right)\right]\right).$$

The approximation of an ideal non-relativistic gas, $E=\hbar^{2}k^{2}/2m$, is known to yield excellent results for metals. For semiconductors with a low filling factor in the conduction band, we can use $E=\hbar^{2}k^{2}/2m_{*}$ if we also calculate *μ *and *E*_*F *_from the minimum of the energy band. For high filling factor, we should calculate the chemical potential and Fermi energy for the holes downwards from the maximum of the energy band, of course, to use $E=\hbar^{2}k^{2}/2m_{h}$.

If the density of effectively free charge carriers in a material is small, as in a semiconductor, then the thermal properties of the electrons or holes can be described by a non-degenerate Fermi gas. With the understanding to calculate energies and chemical potentials from the corresponding energy band extremum, the conditions for applicability of a non-degenerate Fermi gas model for the conduction electrons or holes are

$$E_{F}≲k_{B}T\ll -\mathrm{\mu .}$$

This is equivalent to a requirement of low volume density *n*_*d*_ of charge carriers,

$$\begin{array}{c} n_{d}={\left(\frac{\partial p_{d}}{\partial \mu }\right|}_{T,V}=2{\left(\frac{1}{\hbar }\sqrt{\frac{mk_{B}T}{2\Pi }}\right)}^{d}\text{exp}\;\left(\frac{\mu }{k_{B}T}\right)\\ \;\ll 2{\left(\frac{1}{\hbar }\sqrt{\frac{mk_{B}T}{2\Pi }}\right)}^{d}. \end{array}$$

The pressure and energy density of the carriers are then *p*_*d *_=* n*_*d*_*k*_*B*_*T *and

$$u_{d}=\frac{d}{2}p_{d}=\frac{d}{2}n_{d}k_{B}T,$$

and the entropy density is given by a *d*-dimensional Sackur-Tetrode equation,

(15) $$\begin{array}{l} s_{d}={\left(\frac{\partial p_{d}}{\partial t}\right|}_{V,\mu }=n_{d}\left(\frac{d+2}{2}k_{B}-\frac{\mu }{T}\right)\\ \;=n_{d}k_{B}\left[d\cdot \text{ln}\;\left(\frac{2^{1/d}}{n_{d}^{1/d}\hbar }\sqrt{\frac{mk_{B}T}{2\Pi }}\right)+\frac{d+2}{2}\right]. \end{array}$$

The previous remarks apply to a non-degenerate fermion gas. However, the electron gas in metals has high density and is therefore described by a nearly degenerate non-relativistic electron gas:

$$\mu ∼E_{F}\gg k_{B}\mathrm{T.}$$

In that case, the particle density can be expressed asymptotically in *k*_*B*_*T*/*μ *as

$$\;n_{d}≃\frac{2}{\Gamma (d/2)}{\left(\frac{1}{\hbar }\sqrt{\frac{m\mu }{2\Pi }}\right)}^{d}\left[\frac{2}{d}+(d-2)\frac{\Pi^{2}}{12}{\left(\frac{k_{B}T}{\mu }\right)}^{2}\right],$$

and comparison with (14) yields

$$\mu ≃E_{F}\left[1-(d-2)\frac{\Pi^{2}}{12}{\left(\frac{k_{B}T}{E_{F}}\right)}^{2}\right].$$

The energy density and pressure then follow as

$$u_{d}=\frac{d}{2}p_{d}≃\frac{d}{2}n_{d}E_{F}\left[\frac{2}{d+2}+\frac{\Pi^{2}}{6}{\left(\frac{k_{B}T}{E_{F}}\right)}^{2}\right],$$

i.e., the average energy per electron in a *d*-dimensional metal is

$${\overset{Â¯}{E}}_{d}=\frac{u_{d}}{n_{d}}≃E_{F}\left[\frac{d}{d+2}+d\cdot \frac{\Pi^{2}}{12}{\left(\frac{k_{B}T}{E_{F}}\right)}^{2}\right].$$

The specific heat per *d*-dimensional volume follows as

(16) $$\begin{array}{c} c_{V}={\left(\frac{\partial u_{d}}{\partial t}\right|}_{V,N}≃\frac{d}{6}\Pi^{2}k_{B}^{2}\frac{n_{d}}{E_{F}}T\\ \;\;=\frac{\Pi k_{B}^{2}}{3\hbar^{2}}\frac{\text{mT}}{\Gamma^{2/d}(d/2)}{\left(\frac{d\cdot n_{d}}{4}\right)}^{\frac{d-2}{d}}. \end{array}$$

In terms of the average separation $l=n_{d}^{-1/d}$ of the electrons or holes, the dependence of the specific heat on the physical variables can be summarized as

(17) $$c_{V}\propto \text{mT}l^{2-d}.$$

The specific heat is also related to the thermal conductivity. We can write (16) also in the form

$$c_{V}=\frac{d}{3}\Pi^{2}k_{B}^{2}\frac{n_{d}}{mv_{F}^{2}}T,$$

and therefore, the thermal conductivity for collisional relaxation time *τ* can be written as

$$\kappa_{T}=\frac{1}{d}c_{V}v_{F}^{2}\tau =\frac{1}{3}\Pi^{2}k_{B}^{2}\frac{n_{d}\tau }{m}\mathrm{T.}$$

We can use this result to answer the question whether the relation between thermal and electrical conductivity in a metal is affected by the number of dimensions. The electrical conductivity is

$$\sigma =\frac{n_{d}\tau }{m}e^{2},$$

i.e., the basic Wiedemann-Franz law for the nearly degenerate electron gas in metals holds in every dimension with the same Lorentz constant,

$$\frac{\kappa_{T}}{\sigma }={\left(\frac{\Pi k_{B}}{e}\right)}^{2}\frac{T}{3}.$$

### Inter-dimensional effects in interfaces and thin layers

We know that the *d*-dimensional physics described in the previous sections for *d *= 1 or *d *= 2 can only apply to systems where the technologically relevant degrees of freedom, i.e., mostly electrons and holes as carriers of energy, charge and spin, are confined in sufficiently deep potentials to render any transverse excitations negligible. However, states closer to the binding energy of an attractive potential should exhibit intermittent behavior between low-dimensional and three-dimensional behavior. Furthermore, free states near the ionization energy should also still feel the presence of the low-dimensional structure: the influence of low-dimensional physics cannot discontinuously disappear above the ionization energy. An example of a low-dimensional structure is, e.g., an interface of width 2*a*. Electrons may experience a potential energy *V*_0_in the interface, and they might also move with a different effective mass *m*_∗_ in the interface, such that the Hamiltonian for electrons in the presence of the interface has the form

(18) $$\begin{array}{ll} H=\frac{{\vec{p}}^{2}}{2m}\left[1-\Theta (z_{0}+a-z)\Theta (z-z_{0}+a)\right]\\ \;+\Theta (z_{0}\;+\;a-z)\Theta (z-z_{0}\;+\;a)\left(\frac{{\vec{p}}^{2}}{2m_{*}}\;+\;V_{0}\right). & \end{array}$$

We will denote two-dimensional coordinate vectors parallel to the interface with $x_{∥}=(x,y)=x{\vec{e}}_{x}+y{\vec{e}}_{y}$, $\vec{x}=x_{∥}+z{\vec{e}}_{z}$.

We might expect two-dimensional behavior in the limit *a *→ 0 both from the difference of effective mass in the interface and from the interface potential. Indeed, it has been shown that even without a potential difference, the existence of a layer with different effective mass generates Green’s functions in the interface which interpolate between two-dimensional behavior for small distance $\left|x-x^{\prime }\right|$ and three-dimensional behavior for large distance along the interface [4,5,10].

In the following, we will investigate the emergence of quasi two-dimensional behavior from an attractive interface potential *V*_0_ < 0 in the interface, i.e., we assume *m*_∗_ =* m *in (18). An infinitely thin attractive quantum well arises from the potential in (18) if we set $V_{0}=-W/2a<0$ and take the limit *a *→ 0,

$$H=\frac{{\vec{p}}^{2}}{2m}-W\delta (z-z_{0}).$$

The corresponding Schrödinger equation separates and yields three kinds of energy eigenstates. First, we have eigenstates which are moving along the interface,

(19) $$\langle \vec{x}|k_{∥},\kappa \rangle =\frac{\sqrt{\kappa }}{2\Pi }\text{exp}\;\left(ik_{∥}\cdot x_{∥}-\kappa |z-z_{0}|\right),\;\kappa =\frac{m}{\hbar^{2}}W,$$

$$E(k_{∥},\kappa )=\frac{\hbar^{2}}{2m}k_{∥}^{2}-\frac{m}{2\hbar^{2}}W^{2}.$$

We also have free states with odd or even parity under *z *→ 2*z*_0_ −* z*,

(20) $$\langle \vec{x}|k_{∥},k_{⊥},-\rangle =\frac{1}{2{\sqrt{\Pi }}^{3}}\text{exp}\;\left(ik_{∥}\cdot x_{∥}\right)\text{sin}[k_{⊥}(z-z_{0})],$$

(21) $$\begin{array}{l} \;\langle \vec{x}|k_{∥},k_{⊥},+\rangle =\text{exp}\;\left(ik_{∥}\cdot x_{∥}\right)\\ \;\;\;\times \frac{k_{⊥}\text{cos}[k_{⊥}(z-z_{0})]-\kappa \text{sin}[k_{⊥}|z-z_{0}|]}{2\sqrt{\Pi^{3}(\kappa^{2}+k_{⊥}^{2})}}. \end{array}$$

The wave number *k*_⊥_ in (20) and (21) is constrained to the positive half-line *k*_⊥_ > 0, and the energy levels of the free states are

$$E(k_{∥},k_{⊥})=\frac{\hbar^{2}}{2m}\left(k_{∥}^{2}+k_{⊥}^{2}\right).$$

The completeness relation for the eigenstates is

$$\begin{array}{l} \int \;d^{2}k_{∥}\int_{0}^{\infty }\;dk_{⊥}\left(\langle \vec{x}|k_{∥},k_{⊥},-\rangle \langle k_{∥},k_{⊥},-|{\vec{x}}^{\prime }\rangle \right)\\ \left(+\langle \vec{x}|k_{∥},k_{⊥},+\rangle \langle k_{∥},k_{⊥},+|{\vec{x}}^{\prime }\rangle \right)\\ +\int \;d^{2}k_{∥}\;\langle \vec{x}|k_{∥},\kappa \rangle \langle k_{∥},\kappa |{\vec{x}}^{\prime }\rangle =\delta (\vec{x}-{\vec{x}}^{\prime }). \end{array}$$

The energy-dependent Green’s function

$$\begin{array}{c} \langle x_{∥},z|G(E)|x_{∥}^{\prime },z^{\prime }\rangle \equiv \langle z|G(x_{∥}-x_{∥}^{\prime },E)|z^{\prime }\rangle \\ \;\;\;\;\;\equiv -\frac{\hbar^{2}}{2m}\langle z|G(x_{∥}-x_{∥}^{\prime },E)|z^{\prime }\rangle \end{array}$$

of this system must satisfy

(22) $$\begin{array}{c} \left(\Delta +\frac{2m}{\hbar^{2}}\left[E+W\delta (z-z_{0})\right]\right)\langle z|G(x_{∥},E)|z^{\prime }\rangle \\ \;=-\delta (x_{∥})\delta (z-z^{\prime }). \end{array}$$

This equation can be solved analytically using the methods described in [5,6]. The results are conveniently reported in a mixed axial representation

(23) $$\begin{array}{l} \;\langle k_{∥},z|G(E)|k_{∥}^{\prime },z^{\prime }\rangle =\frac{1}{{\left(2\Pi \right)}^{2}}\;\int \;d^{2}x_{∥}\;\int \;d^{2}x_{∥}^{\prime }\langle x_{∥},z|G(E)|x_{∥}^{\prime },z^{\prime }\rangle \\ \;\;\;\;\;\times \text{exp}\;\left[i\;\left(k_{∥}^{\prime }\cdot x_{∥}^{\prime }-k_{∥}\cdot x_{∥}\right)\right]\\ \;\;\;\;\;=\langle z|G(k_{∥},E)|z^{\prime }\rangle \delta \;\left(k_{∥}-k_{∥}^{\prime }\right), \end{array}$$

$$\langle z|G(k_{∥},E)|z^{\prime }\rangle =\int \;d^{2}x_{∥}\;\langle z|G(x_{∥},E)|z^{\prime }\rangle \text{exp}\;\left(-ik_{∥}\cdot x_{∥}\right).$$

The retarded solution of Equation (22) in the representation (23) is

(24) $$\begin{array}{l} \;\langle z|G(k_{∥},E)|z^{\prime }\rangle =\frac{\hbar \Theta (\hbar^{2}k_{∥}^{2}-2\text{mE})}{2\sqrt{\hbar^{2}k_{∥}^{2}-2\text{mE}}}\\ \;\times \left[\text{exp}\;\left(-\sqrt{\hbar^{2}k_{∥}^{2}-2\text{mE}}\frac{\left|z-z^{\prime }\right|}{\hbar }\right)\right)\\ \;+\frac{\hbar \kappa }{\sqrt{\hbar^{2}k_{∥}^{2}-2\text{mE}}-\hbar \kappa -i\varepsilon }\\ \;\left(\times \text{exp}\;\left(-\sqrt{\hbar^{2}k_{∥}^{2}-2\text{mE}}\frac{\left|z-z_{0}|+|z^{\prime }-z_{0}\right|}{\hbar }\right)\right]\\ \;+i\frac{\hbar \Theta (2\text{mE}-\hbar^{2}k_{∥}^{2})}{2\sqrt{2\text{mE}-\hbar^{2}k_{∥}^{2}}}\left[\text{exp}\;\left(i\sqrt{2\text{mE}-\hbar^{2}k_{∥}^{2}}\frac{\left|z-z^{\prime }\right|}{\hbar }\right)\right)\\ \;+\frac{i\hbar \kappa }{\sqrt{2\text{mE}-\hbar^{2}k_{∥}^{2}}-i\hbar \kappa }\\ \;\left(\times \text{exp}\;\left(i\sqrt{2\text{mE}-\hbar^{2}k_{∥}^{2}}\frac{\left|z-z_{0}|+|z^{\prime }-z_{0}\right|}{\hbar }\right)\right]. \end{array}$$

The limit *κ *→ 0 reproduces the corresponding representation of the free retarded Green’s function in three dimensions.

Our result describes the Green’s function for a particle in the presence of the thin quantum well, but for arbitrary energy and both near and far from the quantum well. Therefore, we cannot easily identify any two-dimensional limit from the Green’s function. To analyze this question further, we will look at the zero energy Green’s function *G*(*r*) ≡ 〈*z*_0_|*G*(***x***_∥_,*E *= 0)|*z*_0_〉, *r *= |***x***_∥_|, in the thin quantum well. Fourier transformation of our result (24) yields

(25) $$G(r)=\frac{1}{4\Pi r}-\frac{\kappa }{8}\left[Y_{0}(\kappa r)+H_{0}(\kappa r)\right],$$

where **H**_0_(*κr*) is a Struve function. The Green’s function (25) has the property to approach a linear combination of two-dimensional and three-dimensional Green’s functions at small distances,

(26) $$G(r){|}_{\kappa r<0.5}≃\frac{1}{4\Pi r}-\frac{\kappa }{4\Pi }\left[\text{ln}\;\left(\frac{\kappa r}{2}\right)+\gamma \right],$$

but it is very different from either the two-dimensional or three-dimensional behavior at large distances,

(27) $$G(r){|}_{\kappa r>2.5}≃-\sqrt{\frac{\kappa }{8\Pi r}}\text{sin}\;\left(\kappa r-\frac{\Pi }{4}\right)$$

(see Figure 2).

**Figure 2:**
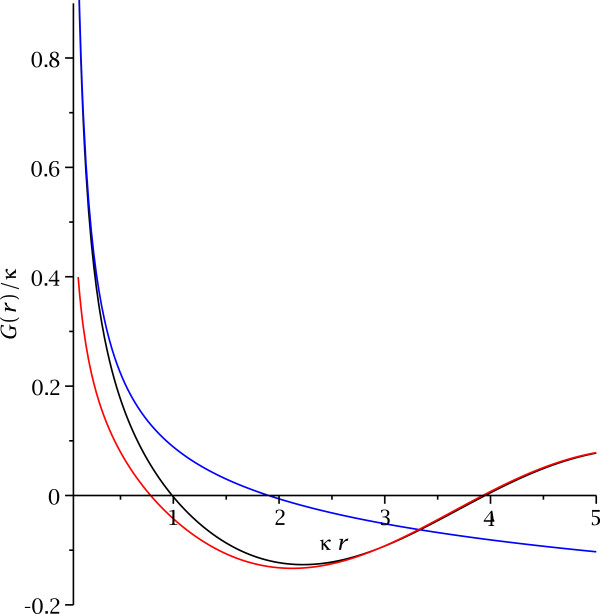
**The zero energy Green’s function in the potential well.** The black curve shows the Green’s function (25). The blue curve is the asymptotic form (26) for small distance and the red curve is the asymptotic form (27) for large distance.

**Figure 3:**
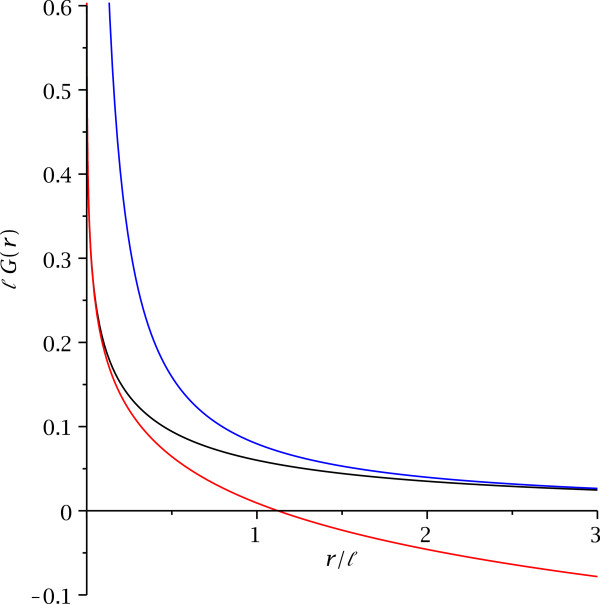
**The zero energy Green’s function in a layer with different effective mass or permittivity.** The blue line is the three-dimensional Green’s function (4*Πr*)^−1^, the black line is the Green’s function (28) in a layer of different effective mass or different permittivity, and the red line is the two-dimensional logarithmic Green’s function *ℓ*·*G*=−[*γ* + ln(*r*/2*ℓ*)]/4*Π*.

It is instructive to compare this to the Green’s function which results from different effective mass or different permittivity in a layer. The corresponding zero energy Green’s function [6]

(28) $$G(r)=\frac{1}{8\ell }\left[H_{0}\;\left(\frac{r}{\ell }\right)-Y_{0}\;\left(\frac{r}{\ell }\right)\right]$$

yields two-dimensional behavior at small distances *r *≪* ℓ *and three-dimensional behavior for large separation *r *≫* ℓ*,

(29) $$r\;\ll \;\ell :G(r)\;=\;\frac{1}{4\Pi \ell }\left[\;-\gamma \;-\;\text{ln}\;\left(\frac{r}{2\ell }\right)\;+\;\frac{r}{\ell }\;+\;O\;\left(\frac{r^{2}}{\ell^{2}}\right)\right],$$

(30) $$r\gg \ell :G(r)=\frac{1}{4\Pi r}\left[1-\frac{\ell^{2}}{r^{2}}+O\;\left(\frac{\ell^{4}}{r^{4}}\right)\right]$$

(see also Figure 3). Here, the length parameter *ℓ *is *ℓ *=* am*/*m*_∗_ for an interface with different effective mass *m*_∗_ for electrons or holes, or *ℓ *=* aε*_∗_/*ε* for an interface with different permittivity *ε*_∗_.

To explore the question of two-dimensional or three-dimensional behavior in the quantum well further, we will look at the density of states in the quantum well.

### Density of states for the thin quantum well

The energy dependent retarded Green’s function is directly related to the density of states in a quantum system. This follows readily from the decomposition of (*E*−*H* + i*ε*)^−1^ in terms of the spectrum *E*_*n *_and eigenstates |*n*,*ν*〉 of *H*,

(31) $$\begin{array}{l} \;G(E)=-\frac{2m}{\hbar^{2}}G(E)=\frac{1}{E-H+i\varepsilon }=\underset{n,\nu }{\sum }\frac{\left|n,\nu \rangle \langle n,\nu \right|}{E-E_{n}+i\varepsilon }\\ \;=P\underset{n,\nu }{\sum }\frac{\left|n,\nu \rangle \langle n,\nu \right|}{E-E_{n}}-i\Pi \underset{n,\nu }{\sum }\delta (E-E_{n})|n,\nu \rangle \langle n,\nu |. \end{array}$$

Here, *ν *is a degeneracy index, and we tacitly imply that continuous components in the indices (*n*,*ν*) are integrated. We include spin in the set of quantum numbers (*n*,*ν*).

To make the connection with the density of states (or number of electronic orbitals) per volume, we observe that this quantity in general can be defined as

(32) $$\varrho (E,\vec{x})=\underset{n,\nu }{\sum }\;\delta (E-E_{n}){\left|\langle \vec{x}|n,\nu \rangle \right|}^{2}.$$

This implies the relation

(33) $$\varrho (E,\vec{x})=-\frac{1}{\Pi }I\langle \vec{x}|G(E)|\vec{x}\rangle .$$

The quantum well at *z*_0_ breaks translational invariance in *z* direction, and we have with equation (33)

$$\begin{array}{l} \varrho (E,z)=\frac{4m}{\Pi \hbar^{2}}I\langle x_{∥},z|G(E)|x_{∥},z\rangle \\ \;\;=\frac{m}{\Pi^{3}\hbar^{2}}I\int \;d^{2}k_{∥}\;\langle z|G(k_{∥},E)|z\rangle , \end{array}$$

where a factor *g *= 2 was taken into account for spin 1/2 states.

If there is any quasi two-dimensional behavior in this system, we would expect it in the quantum well region. Therefore, we use the result (24) to calculate the density of states *ϱ*(*E*,*z*_0_) in the quantum well. Substitution yields

$$\begin{array}{ll} \varrho (E,z_{0})=\frac{m}{\Pi^{3}\hbar^{2}}I\int \;d^{2}k_{∥}\;\langle z_{0}|G(k_{∥},E)|z_{0}\rangle \\ \;\;\;=\frac{m}{\Pi \hbar }\int_{0}^{\infty }\;\text{dk}\;k\;\delta \;\left(\sqrt{\hbar^{2}k^{2}-2\text{mE}}-\hbar \kappa \right)\\ \;\;+\frac{m}{\Pi^{2}\hbar }\Theta (E)\int_{0}^{\sqrt{2\text{mE}}/\hbar }\;\text{dk}\;\frac{k\sqrt{2\text{mE}-\hbar^{2}k^{2}}}{2\text{mE}-\hbar^{2}k^{2}+\hbar^{2}\kappa^{2}}, & \end{array}$$

and after evaluation of the integrals,

(34) $$\begin{array}{l} \;\varrho (E,z_{0})=\Theta (2\text{mE}+\hbar^{2}\kappa^{2})\kappa \frac{m}{\Pi \hbar^{2}}\\ \;\;+\Theta (E)\frac{m}{\Pi^{2}\hbar^{3}}\left[\sqrt{2\text{mE}}\;-\;\hbar \kappa \;\text{arctan}\;\left(\frac{\sqrt{2\text{mE}}}{\hbar \kappa }\right)\right]. \end{array}$$

We can also express this in terms of the free two-dimensional and three-dimensional densities of electron states (cf. (13)),

(35) $$\begin{array}{l} \varrho (E,z_{0})=\kappa \varrho_{d=2}\;\left(E+(\hbar^{2}\kappa^{2}/2m)\right)\\ \;\;+\varrho_{d=3}(E)\left[1\;-\;\frac{\hbar \kappa }{\sqrt{2\text{mE}}}\text{arctan}\;\left(\frac{\sqrt{2\text{mE}}}{\hbar \kappa }\right)\right]. \end{array}$$

Note that *K*_2_ =* E* + (*ℏ*^2^*κ*^2^/2*m*) is the kinetic energy of the particles whose wave functions are exponentially suppressed perpendicular to the quantum well. We find that these particles indeed contribute a term proportional to the two-dimensional density of states *ϱ*_*d *= 2_(*K*_2_) with their energy *K*_2_ of motion along the quantum well, but with a dimensional proportionality constant *κ *which is the inverse penetration depth of those states. Such a dimensional factor has to be there because densities of states in three dimensions enumerate states per energy and per volume, while *ϱ*_*d *= 2_(*K*_2_) counts states per energy and per area. Furthermore, the unbound states yield a contribution which approaches the free three-dimensional density of states *ϱ*_*d *= 3_(*E*) in the limit *κ *→ 0.

**Figure 4:**
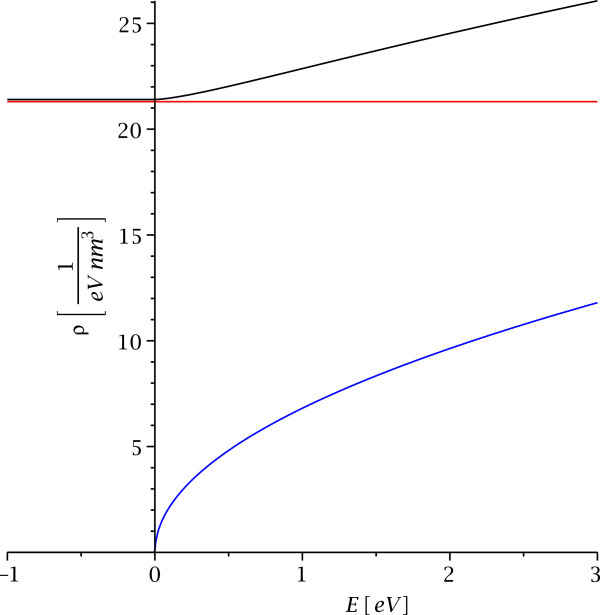
**The density of states in the quantum well.** This displays the density of states in the quantum well location *z *=* z*_0_ for binding energy *B *= 1 eV, mass *m *=* m*_*e*_ = 511 keV/*c*^2^, and energies −*B *≤* E*≤ 3 eV. The red curve is the contribution from states bound inside the quantum well, the blue curve is the pure three-dimensional density of states in absence of a quantum well, and the black curve is the density of states according to equation (34).

**Figure 5:**
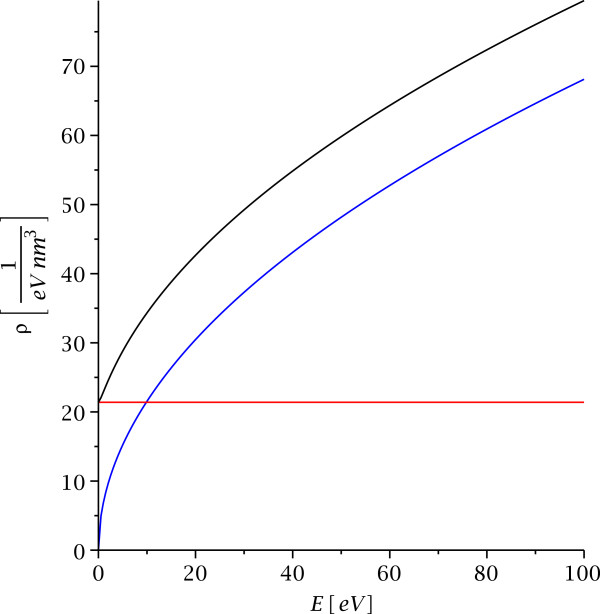
**The density of states (**34**) in the quantum well location *z *= *z***_**0**_** for higher energies *0 ≤ E ≤ 100 *eV.** The binding energy, mass and color coding are the same as in Figure 4. The full density of states (34) approximates the three-dimensional $\sqrt{E}$behavior for energies *E *≫* B*, but there remains a finite offset compared to *ϱ*_*d *= 3_ due to the presence of the quantum well.

The density of states in the quantum well region is displayed for binding energy *B *=* ℏ*^2^*κ*^2^/2*m *= 1 eV, mass *m *=* m*_*e*_= 511 keV/*c*^2^, and different energy ranges in Figures 4 and 5. The density of states shows two-dimensional behavior for energies below the threshold where the electrons or holes can leave the quantum well and a linear combination of a two-dimensional term and three-dimensional term (with a correction factor) for energies above the threshold. This is again very different from the corresponding behavior of electrons or holes which move with different effective mass in a layer. In that case, the density of states in the layer approaches three-dimensional behavior for small separation and two-dimensional behavior for large separation [6] (see in particular Equations (11) to (13) and Figure 1 in [6]).

Integration of *ϱ*(*E*,*z*_0_) yields the relation between the Fermi energy and the particle density in the quantum well. We find for −*ℏ*^2^*κ*^2^/2*m *≤* E*_*F*_ ≤ 0 the two-dimensional area density for maximal kinetic energy *K*_2,*K*_ =* E*_*F*_ + (*ℏ*^2^*κ*^2^/2*m*) along the barrier, but rescaled with the inverse transverse penetration depth *κ* which converts it into a three-dimensional particle density,

(36) $$n(z_{0}){|}_{-B<E_{F}<0}=\frac{\kappa m}{\Pi \hbar^{2}}\left(E_{F}+\frac{\hbar^{2}\kappa^{2}}{2m}\right)=\kappa n_{2}{|}_{E_{2,F}=K_{2,F}}.$$

The result for *E*_*F *_> 0 is a combination of the scaled two-dimensional particle density (36) and the three-dimensional free particle density *n*_3_ with additional correction terms,

(37) $$\begin{array}{l} \;n(z_{0}){|}_{E_{F}>0}=\frac{\kappa }{2\Pi^{2}\hbar^{2}}\\ \;\times \left[\hbar \kappa \sqrt{2mE_{F}}-\left(\hbar^{2}\kappa^{2}+2mE_{F}\right)\text{arctan}\;\left(\frac{\sqrt{2mE_{F}}}{\hbar \kappa }\right)\right]\\ \;+\frac{\kappa m}{\Pi \hbar^{2}}\left(E_{F}+\frac{\hbar^{2}\kappa^{2}}{2m}\right)+\frac{1}{3\Pi^{2}}{\left(\frac{\sqrt{2mE_{F}}}{\hbar }\right)}^{3}, \end{array}$$

(see Figure 6).

**Figure 6:**
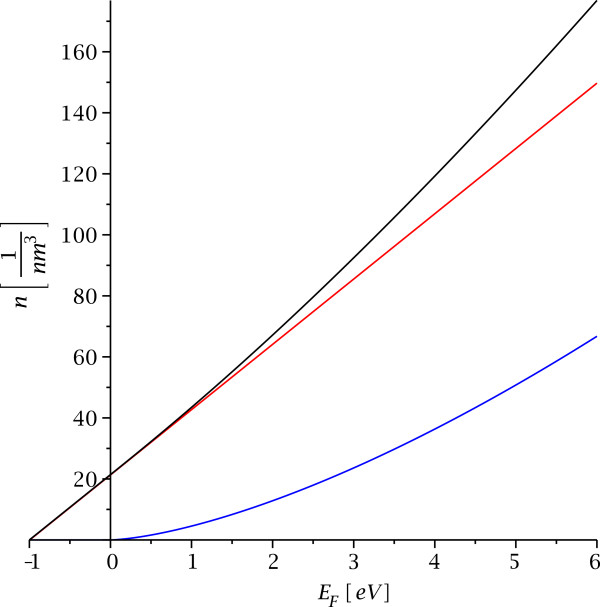
**The relation between particle density and Fermi energy.** This displays the relation between particle density and Fermi energy in the quantum well for binding energy ***B *= 1 eV**, mass ***m *=* m***_***e ***_**= 511 keV/*c***^**2**^, and **−*B *≤* E***_***F ***_**≤6 eV**. The red curve is the contribution from states bound inside the quantum well, the blue curve is the pure three-dimensional density of states in absence of a quantum well, and the black curve is *n*(*E*_*F*_) according to Equations (36) and (37).

The asymptotic form for $E_{F}\gg \hbar^{2}\kappa^{2}/2m$ is given by the three-dimensional density *n*_3_ plus sub-leading correction terms,

(38) $$\begin{array}{l} \;n(z_{0}){|}_{E_{F}\gg B}≃\frac{1}{3\Pi^{2}}{\left(\frac{\sqrt{2mE_{F}}}{\hbar }\right)}^{3}\\ \;\;\;+\frac{\kappa m}{2\Pi \hbar^{2}}\left(E_{F}+\frac{\hbar^{2}\kappa^{2}}{2m}\right)+\frac{\kappa^{2}}{\Pi^{2}\hbar }\sqrt{2mE_{F}}. \end{array}$$

We can also derive these results directly from the energy eigenstates (19 to 21) and the definition (32). However, the derivation from the Green’s function (24) confirms that this is indeed a correct dimensionally hybrid Green’s function which yields inter-dimensional effects.

Not surprisingly, comparison of the relation between Fermi energy and density of fermions for the quantum well with the corresponding results for a layer of different effective mass [6] confirms again that the effective mass layer exhibits separation of two-dimensional behavior for small lengths/high energies and three-dimensional behavior for large lengths/small energies, whereas the quantum well yields a linear combination of two-dimensional and three-dimensional terms for small lengths/high energies.

## Conclusions

The thin quantum well is certainly one of the most important model systems for low-dimensional structures in nano-science and technology. We have found that the Green’s function of this system resembles a linear combination of two-dimensional and three-dimensional terms at small distances but exhibits oscillatory behavior at large distances. Furthermore, the local density of states and the relation between particle density and Fermi energy in the quantum well show two-dimensional behavior for Fermi energies below the threshold for scattering out of the quantum well and a linear combination of two-dimensional and three-dimensional behavior plus correction terms above the threshold. This behavior is very different from the behavior of charge carriers which move with different effective mass in a layer: in that case, the analysis in [6] had shown that the system exhibits two-dimensional behavior at small distances and high energies, and three-dimensional behavior at large distances and low energies. The morale of the combination of the present results with the results of [6] is that if we wish to explicitly see transitions between two-dimensional and three-dimensional behavior in a system, then we should look for systems where the interface primarily affects the kinetic terms of fermions through a difference of effective mass between bulk and layer, or the kinetic terms of photons through a difference of permittivity between bulk and layer.

## Competing interests

The author declares that he has no competing interests.
